# Relationship Between Wettability and Lubrication Characteristics of the Surfaces of Contacting Phospholipid-Based Membranes

**DOI:** 10.1007/s12013-012-9437-z

**Published:** 2012-10-26

**Authors:** Zenon Pawlak, Aneta D. Petelska, Wieslaw Urbaniak, Kehinde Q. Yusuf, Adekunle Oloyede

**Affiliations:** 1 Tribochemistry Consulting, Salt Lake City, UT 84117 USA; 2 Biotribology Laboratory, University of Economy, Garbary 2, 85-229 Bydgoszcz, Poland; 3 Institute of Chemistry, University in Bialystok, Al. J. Pilsudskiego 11/4, 15-443 Białystok, Poland; 4 Faculty of Mathematics, Physics and Technical Sciences, Kazimierz Wielki University, Chodkiewicza 30, 85-867 Bydgoszcz, Poland; 5 School of Chemistry, Physics and Mechanical Engineering, Queensland University of Technology, GPO Box 2434, Brisbane, QLD 4001 Australia

**Keywords:** Joint lubrication, Phospholipids, Surface hydrophobicity, Surface amorphous layer, Wettability

## Abstract

The wettability of the articular surface of cartilage depends on the condition of its surface active phospholipid overlay, which is structured as multi-bilayer. Based on a hypothesis that the surface of cartilage facilitates the almost frictionless lubrication of the joint, we examined the characteristics of this membrane surface entity in both its normal and degenerated conditions using a combination of atomic force microscopy, contact angle measurement, and friction test methods. The observations have led to the conclusions that (1) the acid–base equilibrium condition influences the lubrication effectiveness of the surface of cartilage and (2) the friction coefficient is significantly dependent on the hydrophobicity of the surface of the tissue, thereby confirming the hypothesis tested in this paper. Both wettability angle and interfacial energy were obtained for varying conditions of the cartilage surface both in its wet, dry and lipid-depleted conditions. The interfacial energy also increased with mole fraction of the lipid species reaching an asymptotic value after 0.6. Also, the friction coefficient was found to decrease to an asymptotic level as the wettability angle increased. The result reveal that the interfacial energy increased with pH till pH = 4.0, and then decreased from pH = 4.0 to reach equilibrium at pH = 7.0.

## Introduction

The presence of phospholipid (PL) bilayers on the surface of articular cartilage provides characteristics that are well adapted to wet and relatively dry conditions. This smart surface characteristic creates a hydrophobic–hydrophilic balance resulting in a functional hydrophilic surface in the intact joint. One of the quantitative indicators of surface tribochemical properties is its hydrophobicity. This is measured as wettability or the contact angle between a drop of water and the surface. A poor lubrication in animal joints, particularly on the articular surface of cartilage can be attributed to deterioration in the surface hydrophobicity, where the wettability or contact angle (θ) changes from 100° to less than 70° [1]. PLs form part of the porous solid matrix and any loss in their quantity either through abrasion or disease has been reported to change the wettability [2, 3] which in turn, results in a change of the frictional properties of the surface [1, 3, 4]. These PLs are also present in the synovial fluid (SF) which has a pH of ~7.4 [5]. The wetted surfaces of the PL membranes are negatively (−PO_4_^−^) charged.

**Fig. 1:**
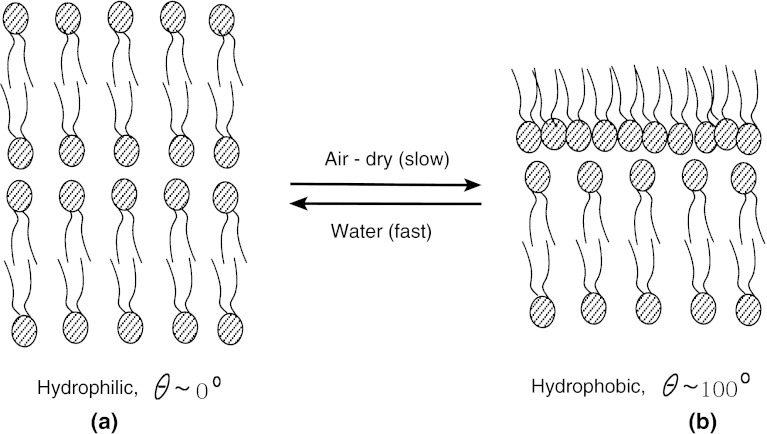
The smart surface constitution of superficial phospholipid bilayer of articular cartilage in: **a** water and **b** air-dry. A change in surface energy leads to conformational changes in the surface phospholipids from bilayer (hydrophilic) to monolayer (hydrophobic) (see Fig. 6c for details)

In this study, we have assumed Hills’ model of the articular surface of cartilage which describes PLs as the major ‘solid’ component of the lubricant in articulating joints [1, 6, 7], the fluid being pressurized water [8]. Following this model, it can be further argued that the PLs in association with pressurized fluid act as a hydrated semi-solid surfactant facilitating the almost frictionless lubrication of the mammalian joint [9–11]. Biosurface wettability can be measured relative to the differences in the charge density of functional amino (−NH_3_^+^) and phosphate (−PO_4_^−^) groups. In this regard, we note that Hills [12] reported that the interfacial energy and wettability of a surface that is characterized by charged anionic phosphate (−PO_4_^−^) groups are lower than those for dry bilayer PLs surface by activating hydrophobic groups, see Fig. 1 [13–15].

Consequently, in their paper, Hills and Crawford [16] stated that: “this raises the fascinating questions of what substance could have been deposited from SF (and lubricin) to render the surface so hydrophobic and yet simultaneously act as such a very effective boundary lubricant”. The lubrication system proposed by Hills is one which PL molecules are arranged in strongly cohesive sheets resembling natural membrane. The stacking of three to seven lipid bilayers separated by aqueous layers has been demonstrated for most biological rubbing surfaces [17]. It is now a well-developed opinion that the gradual removal or damage to this superficial PL layer or surface amorphous layer (SAL) is a key component of osteoarthritis [1, 4]. Specifically, the SAL comprises lamellar bodies, charged macromolecules (lubricin, hyaluronan), and negatively charged PL micelles [17–20] that creates the hydrophobic condition which, in the presence of water, provides a most astonishing low sliding friction [1], see Fig. 1 [7].

We hypothesize that the fundamental mechanism underlying the frictionless lubrication of articulating mammalian joints is the capacity of the ‘superficial phospholipid layer’ to transform itself, through wetting or creation of a surface film from hydrophobic to hydrophilic condition. In other words, the function of the PLs is to create surface hydrophobicity which is the primary requirement for generating the basic surface “wetness” to provide a water-facilitated and essentially frictionless surface (SAL). It can be argued that this characteristic is what is lost or reduced when articular cartilage degrades.

Based on experiments on engineering tribopairs, it has become increasingly apparent that the SF alone cannot facilitate effective lubrication [21]. Cartilage literature has established that the SF in the joint is more of a vehicle for transporting PL molecules to the cartilage–cartilage contact site. The major macromolecules of the SF are lubricin and hyaluronic acid (HA). These constituents act as carriers for PLs and have been argued as contributory to good lubrication in the presence of liposomes [17, 22].

Electron microscope studies of the SAL structure revealed the presence of lamellated PLs (bilayers) on the articular surface [17]. The work of Sarma and Powell [23] established that phosphatidylcholine and phosphatidylethanoloamine are the major PL species present in articular cartilage SAL membrane. It has also been proposed that the articular joint system functions well with hydrophilic surfaces where the surface charge provides electrostatic ‘double-layer’ repulsion, in addition to the ‘steric’ repulsion of the hydration layer of tightly bound water molecules [12, 24]. The question as to what surface characteristics and mechanisms are responsible for the functional adhesion of the PL micelles and lamellar bodies that support the smooth lubrication of joints still remains largely unanswered.

In this paper, we examine by a couple of correlated experimental methods, such as AFM, tribotesting and surface-tension, together with surface wettability measurements, a complex relationship between wettability and friction–lubrication in pH-dependent conditions. It is argued that the wettability of cartilage significantly depends on the number of PL bilayers acting as solid lubricant; this hypothesis was tested by conducting friction tests with normal and lipid-depleted cartilage samples. AFM imaging and interfacial energy methods were used to examine the multi-bilayer cartilage surface. The main finding relies on disclosing in an experimental, but also comprehensive way, a rule that the tribosystem’s delipidization opposes its well-lubricating conditions, the PLs alone are not capable of doing so unless in the presence of water and other biomolecular components of SF. The PLs, which according to Hills’ biological lubrication model and our lamellar–roller lubrication model constitute the main solid-phase component in the joint tribosystem.

## Experimental

The measurements described below were designed to calculate parameters that were used to characterize the basic nature of the hydrophobicity of layers formed with PLs.

### Pertinent Theory

Young–Dupre equation can be used to calculate the wettability of surfaces. This equation is expressed as

1 $$ \cos \theta = (\gamma_{\text{s}} - \gamma_{\text{SL}} )/\gamma_{\text{L}} $$

where $\gamma_{\text{SL}}$ is the decrease in the interfacial energy between the solid and lubricant, $\gamma_{\text{SL}}$ is the interfacial energy of lubricant containing surfactant, $\gamma_{\text{S}}$ is the interfacial energy between the solid and air. Once (θ) is determined from Eq. (1), its value can be used to evaluate the work of adhesion *W*_adh_, for liquid/solid contacts using,

2 $$ {\text{W}}_{\text{adh}} = \gamma_{\text{L}} (1 + \cos \theta ) $$

It should be noted that an equilibrium value of contact angle is used in calculations. Following Eqs. (1) and (2), if we have two different equilibrium contact angles for water on two different surfaces, the higher angle will correspond to a more hydrophobic surface and a lower work of adhesion. The work of adhesion is an accurate reflection of the wettability of a surface, which in turn correlates to the frictional properties of such a surface. It has been reported that under the boundary lubrication regime, a low work of adhesion implies a low frictional contact [25].

In this research, we have employed bovine cartilage to obtain insight into the role of the PLs on the surface of cartilage, with specific emphasis on the mechanisms facilitating the frictional properties of articulating mammalian joints. The method consists of imaging and characterizing the surface of normal intact and progressively delipidized cartilage, while also measuring the coefficient of friction at the interfaces of these materials when in contact under load. We also present the evidence that the superficial PL layer covering the articular surface of cartilage has a primary function of creating a hydrophobic surface whose wetting properties, and hence, control of interfacial properties varies with pH values. Thus the experiments were conducted in solutions with pH in the range 1.5–9.0. The coefficients of friction and interfacial energy versus mole fraction of [phosphatidylcholine (PC) + phosphatidylethanoloamine (PE)] and [PE + cholesterol (CH)] of the contacting membranes were also measured.

### Measurement of Interfacial Energy

The interfacial energy γ of the lipid bilayer was determined by measuring the radius of curvature *r* of the convex surface formed when a pressure difference *p* is applied on its sides. This was based on Young’s and Laplace’s equation,

3 $$ 2\gamma = r\Delta p, $$

where the components of Eq. (3) are as defined above.

The dependence of interfacial energy on the pH of the electrolyte solution has the form [26].

4 $$ \gamma = \gamma_{ \hbox{Max} } + 2sRT\ln \left( {\sqrt {\frac{{K_{a} }}{{K_{b} }}} + 1} \right) - sRT\ln \left[ {\left( {\frac{{K_{a} }}{{a_{{H^{ + } }} }} + 1} \right)\left( {\frac{{a_{{H^{ + } }} }}{{K_{b} }} + 1} \right)} \right] $$

where *K*_a_ and *K*_b_ are acid and base equilibrium constants, respectively, *s* (mol m^−2^) is the surface concentration of PLs, $a_{{{\text{H}}^{ + } }}$ is the hydrogen ion (H^+^) concentration, *R* is the gas constant, *T* is the temperature, and γ_max_ is the maximum interfacial energy of the lipid membrane. The surface area occupied by the PL molecules was calculated from the surface concentration according the equation presented below:

5 $$ s = \frac{1}{{N_{\text{A}} \cdot A}} $$

where *s* is the surface concentration of PLs, *A* is the surface area occupied by the PL molecules, and *N*_A_ is the Avogadro constant.

**Fig. 2:**
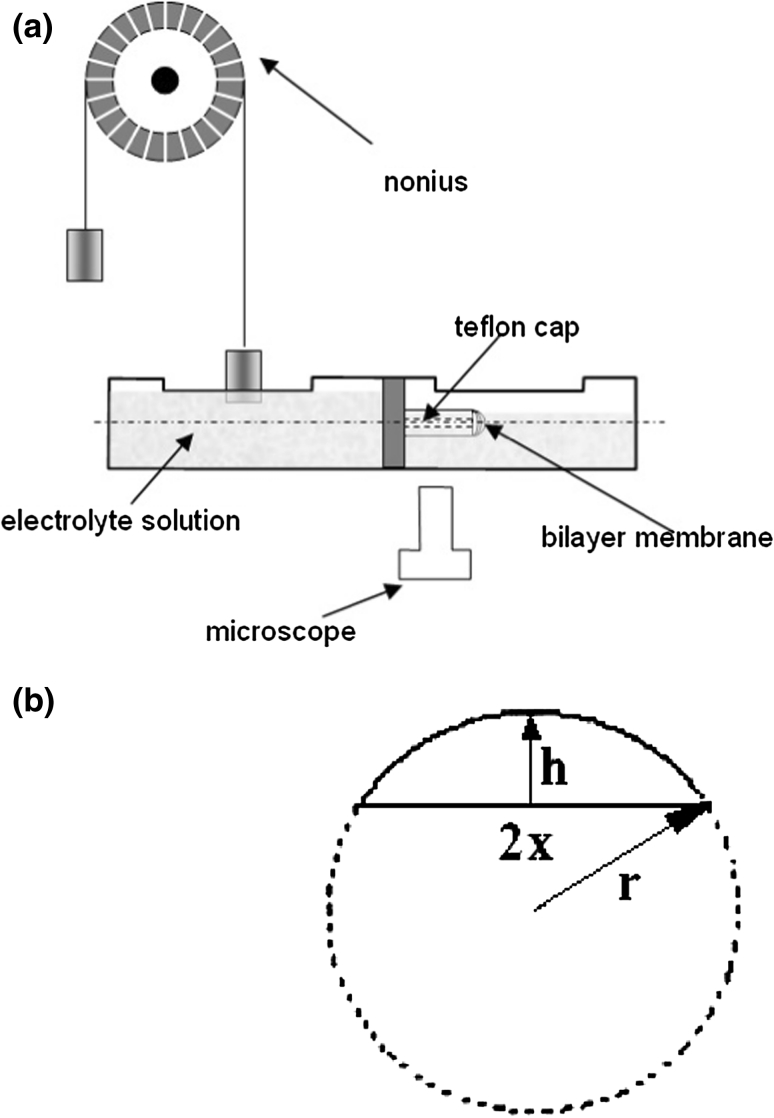
**a** Schematic representation of the apparatus used for measuring interfacial energy. **b** A diagram of the radius of curvature calculation, where *r* is the radius of curvature, *x* is the radius of the Teflon cap and h is the convexity of the lipid membrane

Figure 2a presents the apparatus used to measure the interfacial energy of the lipid membranes in accordance with the earlier work described by Petelska and Figaszewski [27]. The equipment contains two glass chambers separated by a mount holding a 1.5-mm diameter circular Teflon element axially pierced by a small orifice. The membranes were formed in accordance with Mueller–Rudin method [28] on the flat end of the Teflon element. Both chambers were filled with an electrolyte solution. The membrane-forming solution was introduced to the flat wall of the Teflon element using a micropipette, and pressure was applied to the left chamber using a manometer.

The convexity of the lipid membrane cap was measured to a 0.05 mm precision using a microscope equipped with a scale in its objective lens. The radius of curvature, r, was determined using this value and the diameter of the Teflon element which corresponds to the diameter of the lipid cap and convexity of two lipid membranes was also measured as presented in Fig. 2b [29]. The radius of curvature was calculated from equation $= \frac{{x^{2 } + h^{2} }}{2h}$. We measured the radius of curvature of bilayer lipid membrane and the overpressure provoking the membrane convexity. The interfacial energy was calculated from the radius of curvature and pressure difference values according to Young and Laplace’s equation (3).

The interfacial energy was measured on a freshly created lipid bilayer membrane 12–15 times. For each membrane, about 10 instrument readings of the lipid spherical cap diameter formed by the pressure difference applied on both sides were obtained. Measurements with preparation of the electrolyte solution were made 2–3 times in order to test the repeatability of these experiments.

Phosphatidylcholine (PC, 99 %) and phosphatidylethanoloamine (PE, 99 %) and cholesterol (CH, 99 %) produced by Fluka were used. The solution used to form the model membrane contained 20 mg/ml of lipid in *n*-decane. The lipid was dissolved in chloroform to prevent oxidation; the solvent was evaporated in an atmosphere of argon, and the residue was dissolved in *n*-decane, which had been additionally purified by distillation, yielding ε = 1.99 (293.15 K).

The buffer solutions covering the pH range 1.5–9.0 were prepared according to Britton and Robinson [30]. They were used by adding 0.2 M sodium hydroxide to 100 cm^3^ of a solution of the composition: 0.04 M acetic acid (80 %, produced by POCh), 0.04 M phosphoric acid (POCh) and 0.04 M boric acid (POCh). The pH of the buffer was adjusted to a suitable value using sodium hydroxide solution (at 291.15 K). The aqueous solution of 0.1 M KCl (pH 6.90) used for the measurements of interfacial energy of two components (PC–PE) and (PE–CH) membrane was prepared using triply distilled water and KCl (POCh). The pH of the electrolyte was carefully controlled during the measurements.

The articular cartilage specimens were collected from the bovine knees of age 15–20-month old. Osteochondral plugs of 5- and 10-mm diameter were harvested from lateral and medial femoral condyles using a circular stainless steel cutter. The cartilage discs were cut into 3-mm plugs with underlying bone. Two types of samples were tested: untreated bovine cartilage and bovine cartilage treated with lipid rinsing solution to remove the lipids from the surface of the cartilage. After preparation, the specimens were stored at 253.15 K in saline of 0.15 M NaCl (pH = 6.9), and fully defrosted prior to testing. The cartilage discs were then glued to the disc and pin stainless steel surfaces, and lubrication tests were conducted.

### Delipidization Process for Cartilage Surface

The removal of lipids from the superficial layer of cartilage was gradual, and was in accordance with the delipidization procedure described elsewhere [17] using Folch reagent [i.e. a mixture of chloroform/methanol (2:1)] [31]. Briefly, the cartilage samples were immersed in a fat solvent (2:1 chloroform/methanol, v/v) for 1, 3 and 21 min, taking care to maintain the same meniscus. Reducing the time to 15 min was sufficient to remove the PLs from the SAL [2]. After each extraction, sample was placed in saline solution for 15 min for rehydration and to remove organic solvent left on the surface of the cartilage. Following this, the samples were mounted on the atomic force microscope and imaged as described below. These samples were used in the following experiments: surface wettability and lubrication experiments.

### Atomic Force Microscope Analysis of Cartilage Surface

**Fig. 3:**
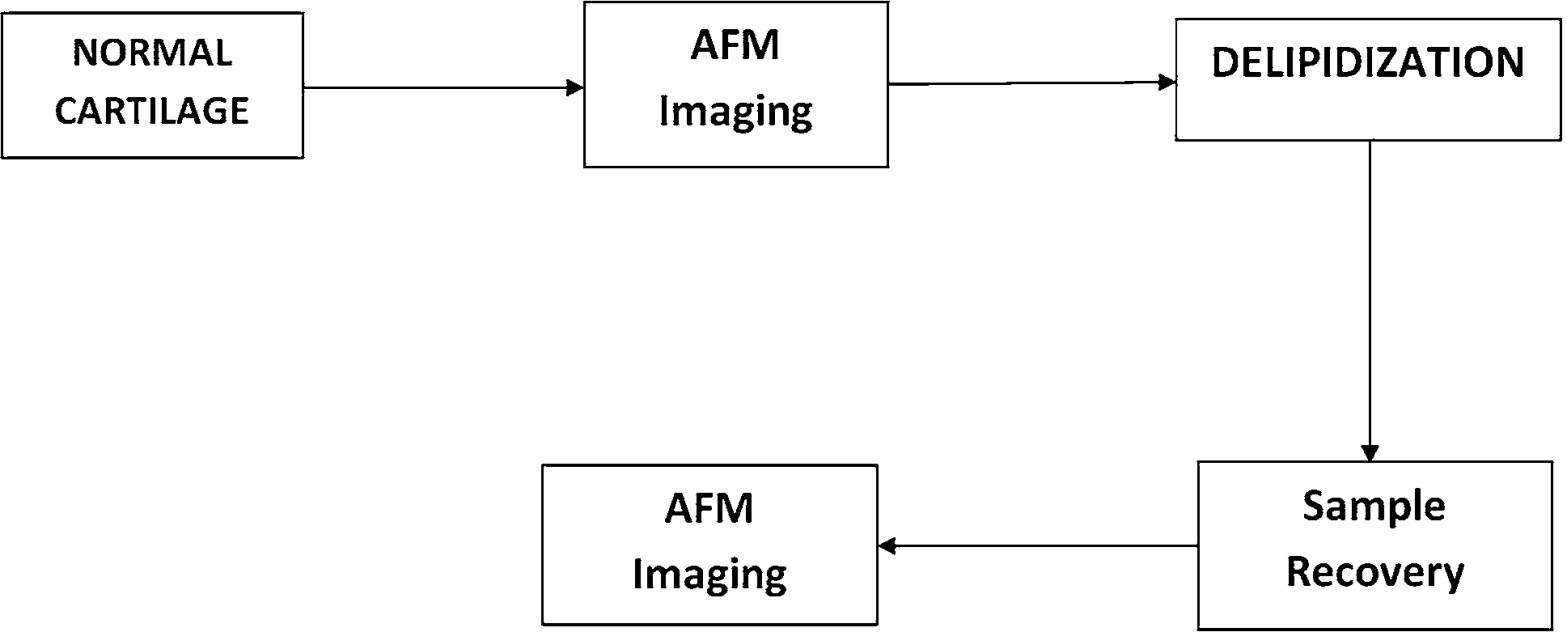
Schematic flowchart of the AFM imaging of normal and delipidized cartilage

Both the normal intact and delipidized surfaces of articular cartilage were characterized with the atomic force microscope. Measurements were conducted with the samples immersed in saline in accordance with the protocol used previously [19, 32, 33]. Osteochondral plugs 3 mm × 3 mm with full thickness articular cartilage that are still attached to their underlying bones (10 samples per patella) were imaged. The bone sublayer was dried with a paper towel and glued onto a Petri dish (1.5 cm in diameter) using a two-sided adhesive tape and a fast-drying Loctite^®^ 454 glue. The normal and delipidized surface characterization was conducted on the same samples, where the lipid removal was done subsequent to the imaging of the normal intact specimen. The surface imaging protocol was conducted to demonstrate the effect of delipidization on the surface amorphous lipid layer of cartilage and to quantify the surface properties of the specimens whose wettability and other properties are sought in this study. The experimental protocol for the AFM imaging of samples is presented schematically in the flow chart (Fig. 3).

A two-sided adhesive tape was used to fix the Petri dish–cartilage specimen onto the sample holder of the AFM. During gluing, the articular surface was moistened with drops of PBS to keep the surface intact. The sample was then submerged in PBS by pipetting PBS into the Petri dish containing the sample to create a liquid environment for AFM measurements. To preserve the integrity of the samples, all experiments were conducted in liquid environment containing PBS using the SMENA^®^ head of the NT-MDT P47 Solver scanning probe microscope (SPM) (NT-MDT, Russia). The imaging was performed with very soft triangular cantilevers (spring constants of between 0.05 and 0.10 kN/m) carrying contact tips (Veeco probes, California, USA). After mounting the specimen and setting up the AFM, the instrument was allowed to undergo thermal relaxation for 30 min, and to ensure that the drift of the cantilever deflection angle was minimized before imaging in accordance with [19, 34].

AFM images were obtained along the 2D planes of the articular surfaces of over 180 samples from 20 normal intact joints, out of which 20 samples where used in this study. Topographical and deflection images were simultaneously captured with height and deflection signals, respectively. In order to obtain high resolution image, the deflection signal was minimized by optimizing scanning parameters such as feedback gain, set-point and scanning speed/frequency. Also, the trace and retrace signals were continuously monitored with the oscillograph to ensure that they were tracking each other. During the entire imaging process, the trace and retrace signals looked similar.

### Contact Angle Measurement

The contact angle between the liquid and tested cartilage was measured using a KSV CAM100 tensiometer. In our experiment, the liquid used was saline and the contact angle measured was that between a droplet of saline and a given cartilage surface. Measurements of contact angle as a function of time were carried out over a duration of 60 min for the normal sample. The measurements of contact angle for delipidized cartilage samples were determined only in dry-air state. The tests were conducted at an ambient laboratory temperature (295.15 K) and relative humidity (HR ~ 45 %). Five tests were performed on each specimen and each set up.

The surface wettability of the cartilage in the articulating joint was measured from its fresh condition to progress exposure to air. After measuring the contact angle for the fresh joint (immediately upon opening the articular cavity), the contact angle was then measured after about 10 min, when the excess surface moisture has been reduced by evaporation by depositing a drop of 0.15 M saline solution. This measurement was repeated every 7 to 15 min from thereon, with a new drop of liquid at different regions on the surface of the same cartilage until a time of 120 min.

### Friction Experiments

The coefficient of friction (*f*) was measured at room temperature using a sliding friction tester pin-on-disc tribotester manufactured by ITeR, Poland. The friction measured was that between two discs of cartilage soaked in saline (lubricating fluid) under a given load, sliding velocities and time. The friction test was conducted at a low speed of 1 mm s^−1^ for 10 min under an applied load of 15 N (1.2 MP), which corresponds to the physiological lubrication condition [35]. The cartilage samples were left for one hour in saline, before each test. Friction tests on delipidized samples were performed under the same conditions as described above. Surfaces were placed in contact and the friction was measured over a period of 10 min. The results of (*f*) as a function of time are given in Fig. 6d for the cartilage–cartilage pairs of normal and delipidized surfaces. In each of the friction pairs, we observed an increase in the friction coefficient, and an increase in the real contact area between the rubbing parts with time. The friction force at the interface was evaluated as a product of contact area and shear stress. A total number of five tests were conducted using fresh samples for each experimental set up.

## Results and Discussion

### Atomic Force Microscopy Characterization

The results obtained from atomic force microscopy (AFM) imaging and characterization of the surface of normal intact and delipidized articular cartilage specimens are presented in this section.

**Fig. 4:**
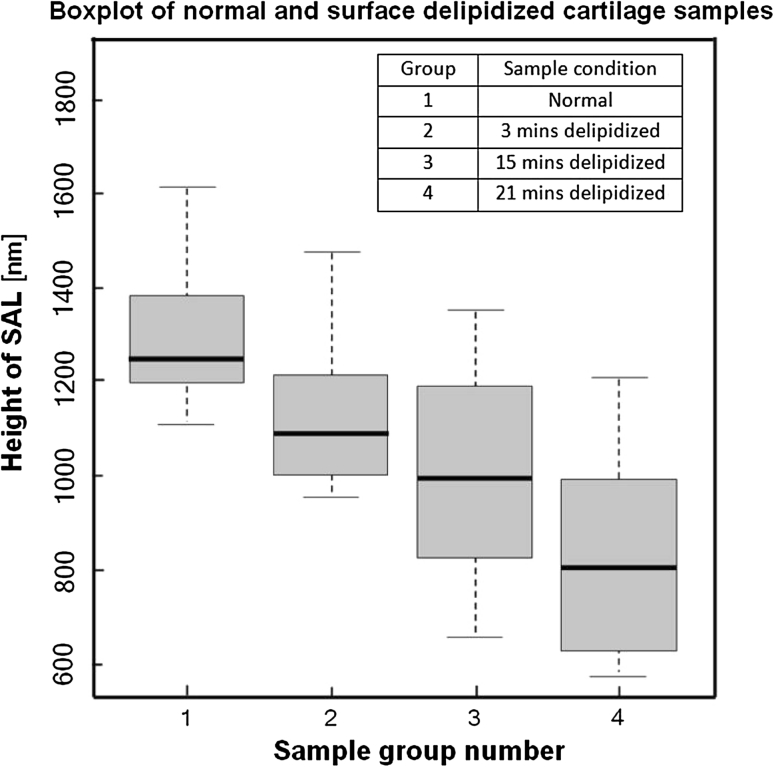
Variation of surface lipid lost (height of SAL, nm) with time following delipidization with chloroform:methanol (2:1). Normal intact (group 1); 3 min delipidization (group 2); 15 min delipidization (group 3); and 21 min delipidization (group 4)

Figure 4 shows the box plot of the height of the SAL of normal intact cartilage and cartilage whose surface has been subjected to different delipidization times in chloroform:methanol (2:1). In each of the delipidization group, we observed a decrease in the heights of SAL with time of exposure in lipid rinsing solvent.

**Fig. 5:**
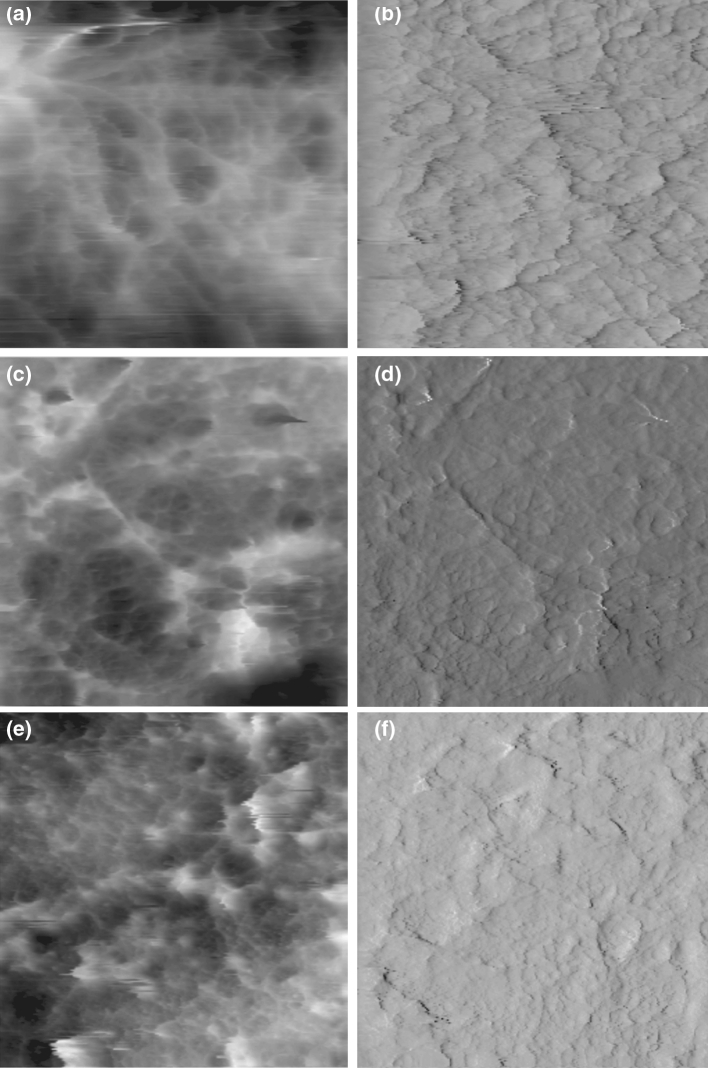
Topographical (**a**, **c**, **e**) and deflection (**b**, **d**, **f**) 2D images of articular cartilage surface (frame size: 8 × 8 μm). Normal articular surface (**a**, **b**); after 3 min delipidization in chloroform/methanol (**c**, **d**); **b** and after 21 min delipidization in chloroform/methanol (**e**, **f**)

Figures 5a and b show the 2D topographical (a) and deflection (b) images acquired simultaneously for normal intact articular cartilage, respectively. The figures reveal that a normal cartilage is covered by a non-fibrous layer of organized surface lipid structure. Hills [17, 36] described this structure as oligolamella layer formed by the surface active phospholipids (SAPL).

Figure 5c and d show the 2D topographical (c) and deflection (d) images of the surface of articular cartilage exposed to chloroform:methanol (2:1) for 3 min. Figures 5e and f on the other hand show the 2-D topographical (c) and deflection (d) images of the articular surface after 21 min exposure in lipid rinsing solvent. Rinsing of the surface of normal cartilage with lipid rinsing agent almost completely removed the organized SAL but no fibre structure was observed in the subsurface layer.

### Interfacial Energy Forces on Phosphatidylethanolamine Membrane

**Fig. 6:**
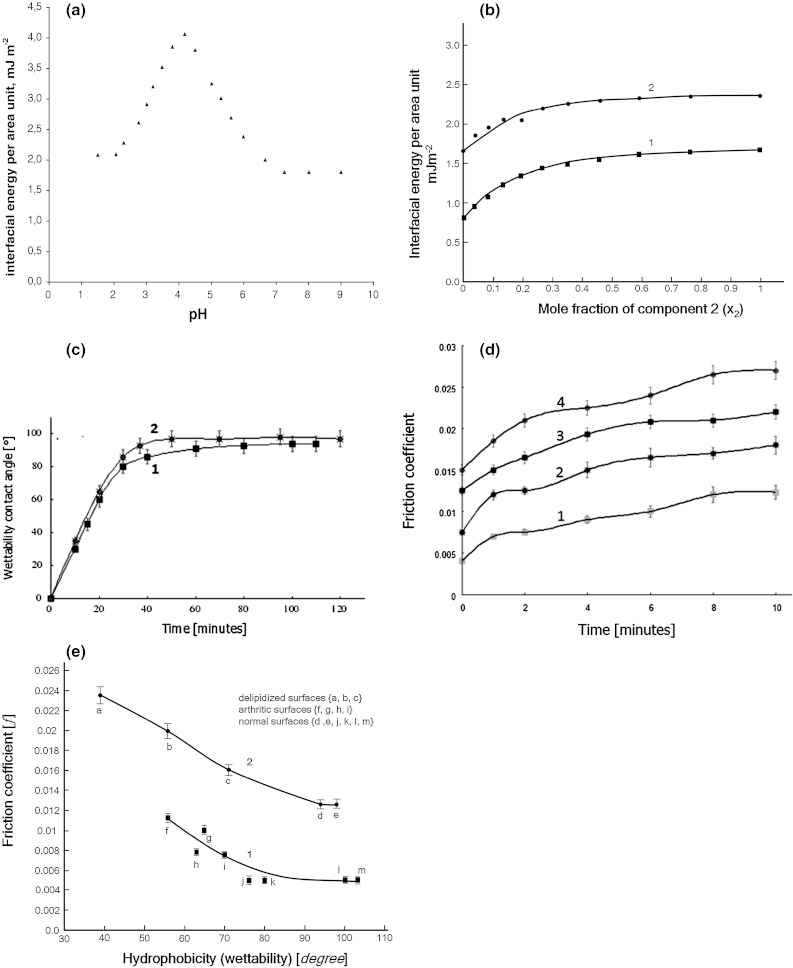
**a** Interfacial energy of bilayer formed by PE as a function of pH with a maximum value (γ_max_) = 4.08 ± 0.12 mJ m^−2^, for Ip, pH = 4.20, and (γ) = 1.80 ± 0.10 mJ m^−2^ for pH = 7.40, and the acidity constants of amino group (−NH_3_^+^), p*K*a = 8.02 and phosphate group (−PO_4_H), p*K*a = 2. 42. **b** Interfacial energy as a function of the mole fraction (*x*_2_) for the phosphatidylcholine–phosphatidylethanolamin (*x*_2_) membrane (I) and the phosphatidylethanolamine–cholesterol (*x*_2_) membrane (II) in 0.1 M aqueous potassium chloride solution at pH 6.90. **c** The wettability contact angle of saline drops on cartilage samples as a function of air-drying time (*1*) joint was opened and air-dried, (*2*) joint was dabbed in saline 60 min and air-dried (*n* = 5, error bars = 95 % confidence limit). **d** Friction coefficient versus time for the cartilage–cartilage pair normal (*curve 1*) and delipidized surfaces measured in saline solution (1, 3 and 21 min): *curves 2, 3 and 4*, respectively; (*n* = 5, error bars = 95 % confidence limit).**e** Coefficient of friction (*f*) versus hydrophobicity (wettability) of various biosurfaces: normal cartilage and arthritic surfaces, *curve 1*, (*square*) [3], and gradually delipidized cartilage surfaces measured in saline solution, curve 2, (*circle*), (*n* = 5, error bars = 95 % confidence limit). Articular surface contact angle (°) of normal cartilage: 103° point m; bovine patella 100.1° point *l*; human knee 79.7° point *k*; hip 76.3° point *j* [3]. Arthritic surface: cartilage 65° point g; bovine patella 70° point *i*; human knee 63 point *h*; hip 56.3° point *f* [3]. Delipidized cartilage bovine knee surface wettability contact angle (°) after: 1 min 71° point *a*; 3 min 56° point *b*; and 21 min 39° point *c*. Normal bovine knee contact angle (°) was 93° point *d*; and 98° point *e*

The effect of pH on interfacial energy of phosphatidylethanolamine (PE) lipid membrane was studied for values of aqueous electrolyte solution with pH of 1.5 to 9.0 at room condition, and their interfacial energy was calculated using Eq. (4) with the results presented in Fig. 6a. The maximum interfacial energy (γ_max_) obtained was 4.08 mJ m^−2^, with a surface concentration of PE of 2.21 × 10^−6^ mol m^−2^ at pH = 4.2. The surface area occupied by a PE molecule was 75 Å^2^. In comparison, under the same conditions, the reported interfacial energy for PC (large hydrophilic head) and phosphatidylserine were 3.53 and 2.93 mJ m^−2^, respectively [37].

PE is a weak polyelectrolyte with amine and phosphate functional groups that are affected by solution pH. At solution pH ~ 2, PLs amine group is in the protonated form (−NH_3_^+^), and (−PO_4_H) is in molecular form, and the surface energy is positively charged and low. As the pH of the solution is increased, the amino groups would begin to lose some of their charges −NH_3_^+^ → −NH_2_), leading to an increase in the interfacial energy towards a maximum value; the (−PO_4_H) group would also undergo a partial loss of proton (−PO_4_H → −PO_4_^−^) as demonstrated by Fig. 6a. The maximum point in Fig. 6a is the isoelectric point (Ip).

At this point, the PLs or the membranous surface constituents would carry no net electrical charge (i.e. the negative and positive charges would be equal) [38]. As the pH of the solution is increased, the amino groups would gradually lose their charges, while the (−PO_4_H) groups lose their protons (−PO_4_H → −PO_4_^−^), leading to a negatively charged surface with decreased interfacial energy. It has been reported that there is a relationship between the size of hydrophilic head of lipid and the pH at the Ip; with the Ip occurring at a lower pH for PLs with larger hydrophilic heads [39]. For instance, the Ip of PE obtained in this study is at pH = 4.20, which compares with those obtained in other studies, are at pH = 4.15 for PC [26] and at pH = 3.80 for phosphatidylserine [37].

### Interfacial Energy of PC–PE and PE–CH Membrane Systems

The dependence of interfacial energy of mixed PL membranes were studied as function of the mole fractions *x*_2_ = 0.1, 0.2, 0.3, 0.4, 0.5, 0.6, 0.7, 0.8, 0.9 and 1.0 in the mixed PC–PE (*x*_2_) and PE–CH (*x*_2_) systems. The results are presented in Fig. 6b. The interfacial energy value of the pure PE membrane was found to be 1.67 × 10 mJ m^−2^, while the interfacial energy values for the PC–PE and PE–CH (1:1) complexes were 1.58 × 10 and 2.30 × 10 mJ m^−2^, respectively.

The surface concentrations for PC–PE and PE–CH complexes are equal to 1.08 × 10^−6^ and 1.31 × 10^−6^ mol m^−2^, respectively. Thus it was possible to determine the areas occupied by PC–PE and PE–CH complexes, which were 154 and 127 Å^2^, respectively. The stability constants of the PC–PE and PE–CH complexes are 2.85 × 10^8^ and 4.16 × 10^10^ m^2^ mol^−1^ respectively, as calculated using the procedure of [39]. It can be inferred from these results that the mixture of the PE–CH is more stable than that of the PC–PE complex. CH is universally present in the plasma membrane, and it has the capacity to increase the stiffness of a membrane while decreasing its fluidity [40, 41].

### Wettability and Friction of Normal and Delipidizied Cartilage/Cartilage Tribopairs

This study concerns the hydrophobicity of the cartilage surface to saline and the effect of delipidization on the frictional characteristics of the surface of the tissue. It has been noticed in an earlier study that the nature and amount of moisture content influences the value of contact angle between water droplet and the articular surface of cartilage, and that biological surfaces in contact with air after drying for about 1 h remained stable [42]. In the natural condition when the joint is completely covered with SF, the surfaces of cartilage can be seen to be highly hydrophilic with a contact angle of zero. This condition changes with increasing time of exposure of a fresh joint to the atmosphere. The contact angle values were plotted as a function of time (see Fig. 6c).

The contact angle increased until reaching an asymptotic value of approximately 94^o^ for samples dried in air (curve 1). This plateau region was maintained for at least, 60 min. The experimental curves in Fig. 6c are very similar; plateau corresponds to a contact angle of 98° for sample of cartilage dabbed in saline for 60 min (curve 2). We found that, once exposed to air, the hydrophilic surfaces slowly (during ~60 min) became hydrophobic. This was established by contact angle measurements, and manifested itself by a slowly increasing contact angle with exposure time. Using saline interfacial energy value of 72.5 mJ m^−2^ and a contact angle of 98°, we obtained a mean value of 22.4 mJ m^−2^ interfacial energy for cartilage, which is very hydrophobic for a biological surface. It can be inferred from these results that the initially very hydrophobic surfaces became more hydrophilic (or more adhesive) when they came in contact with water. When a drop of water was placed on a dry l-*a*-dipalmitoyl-phosphatidylethanoloamine (DPPE) bilayer covered mica surface, the contact angle was 112°, a very hydrophobic surface, and θ = 10° and 60°, after 5 s and 100 s exposure to water, respectively [43].

This transition from HL → HB is more likely due to the overturning (flip–flop) of surface PL molecules, resulting in the exposure of their hydrophobic groups [14] as the aqueous fluid film is drying on the surface. At pH 6.9, saline solution–phosphate group (−PO_4_^−^) would interact with water across the interface through dipole–dipole attraction to increase adhesion. The interpretation of the results also requires consideration of the charge density. At pH 6.9, PL membrane is fully charged in solution (−PO_4_^−^) (hydrophilic) and begins to lose its charges in air-dry condition leaving the surface with more hydrophobic character, which causes an increase in contact angle [13–15]. The wetting studies also indicate that the change of the surface from hydrophobic (HB) to hydrophilic (HL) condition was much faster than the reverse transition from HL → HB, probably because the surface groups became immobilized in the absence of water. When present in aqueous media, cartilage can enhance its adhesion by exploiting the increased surface energy as a result of conformational changes in the surface PLs by activating hydrophilic groups [44–46].

Figure 6d represents coefficient of friction vs. time for the cartilage–cartilage pair for normal and delipidized surfaces. The coefficient of friction (*f*) values after 10 min contact: Normal (AC–AC) the range *f* = 0. 004–0.012 curve 1, and in the range *f* = 0.0075–0.018, 0.0125–0.022 and 0.015–0.027 for the delipidized AC–AC pairs, curves 2, 3 and 4, respectively. This reveals the significantly higher friction coefficients between AC–AC pairs in the delipidized conditions relative to those of the normal AC–AC pairs with intact contacting surfaces. These friction tests thus confirm our hypothesis on the relation between the number of PLs bilayers (or hydrophobicity) and friction [2, 35, 47–49].

Furthermore, it has been shown that under air-dry conditions the contact angle for bovine patella (~100°) is higher than that for arthritic human knee (~70°) [3]. This can be converted into interfacial energy or the work of adhesion, $\left( {W_{\text{adh}} = \gamma_{\text{L}} (1 + \cos \theta )} \right)$, thereby demonstrating that this parameter is ~50 mJ m^−2^ lower in the arthritic human knee compared to normal intact bovine patella. Apart from this change in contact angle, this reduction in interfacial energy could provide the explanation for the friction shift shown in Fig. 6e.

Hills [1, 12, 17, 36] and other researchers [19, 20] have revealed that spontaneously formed SALs (in vivo) consists of some PLs bilayers on normal intact cartilage surface. The self-organization process involved PLs vesicles that are attracted to the hydrophilic cartilage surface to form a “hybrid bilayer” [50, 51]. The results of our present study demonstrates the importance of the balance between wettability, electrostatics and lubricant composition, thereby supporting some of the previous hypotheses on the essential conditions for effective surface lubrication in a functional mammalian joint.

We have subsequently attempted to confirm our lubrication hypothesis by friction tests. Figure 6e represents the plot of coefficient of friction (*f*) versus hydrophobicity (wettability) for various biosurfaces: normal intact and arthritic articular cartilage surfaces, curve 1, and delipidized cartilage surfaces, curve 2. The results derived from own experiment are represented by curve 2, while curve 1 presents data from literature for comparison. The frictional characteristics of this extracted cartilage *(f* = 0.0235) were greatly increased ~195 % compared with the un-extracted cartilage (*f* = 0.0120). The increase in the value of *f* is comparable to the value that was obtained by other studies [2, 4, 48, 49, 52]. We therefore conclude that the PLs overlaying the cartilage surface as bilayers, operate indeed as a lubricant facilitating the SAL in providing the lubrication mechanism that lowers the friction between two contacting biosurfaces.

## Concluding Remarks

We have examined natural articular surface covered by PLs bilayers and shown the relation between wettability (hydrophobicity) and friction coefficient. AFM imaging and characterization of the surface of normal intact and delipidized articular cartilage specimens showed greatly accelerated damage to the articular cartilage. Tests were performed to measure the heights of SAL, the wettability of different biosurfaces using cartilage sample that was sequentially delipidized, while also measuring the coefficients of friction of contacting cartilage surfaces. We also showed that there is a link between coefficient of friction and hydrophobicity (wettability) or interfacial energy of lubricated biotribopairs. It has also been demonstrated that the “smart surface” of cartilage is highly hydrophilic when wet and hydrophobic when air-dry. The surface interfacial energy of bilayer membrane/or joint surfaces in acid–base equilibria (pH) significantly influence the effectiveness of aqueous (biological) lubrication. We have shown that surface wettability can be varied by as much as ~40°, in natural joints where the surface friction can be altered by a factor of 10. It can be argued from the results of the experiments involving the laying of PC and PE lipid layers that the number of PLs bilayers influences both wettability and surface friction, which are fundamentally important factors in joint lubrication. This observation lends credence to the assertion of previous studies that the key to understanding the mechanism of joint lubrication lays in obtaining insight into the relationships between the structure and function of surface lipids.
